# Supplementary material for: An adaptive stress response that confers cellular resilience to decreased ubiquitination
Source: Nat Commun. 2023 Nov 14;14:7348. doi: 10.1038/s41467-023-43262-7 (PMC10646096; doi:10.1038/s41467-023-43262-7)
Supplement: Supplementary file 1 — Supplementary Information [file 41467_2023_43262_MOESM1_ESM.pdf]

Supplementary Information

An adaptive stress response that confers cellular resilience to decreased ubiquitination

Liam C. Hunt, Vishwajeeth Pagala, Anna Stephan, Boer Xie, Kiran Kodali, Kanisha Kavdia, Yong-Dong Wang, Abbas Shirinifard, Michelle Curley, Flavia A. Graca, Yingxue Fu, Suresh Poudel, Yuxin Li, Xusheng Wang, Haiyan Tan, Junmin Peng, Fabio Demontis

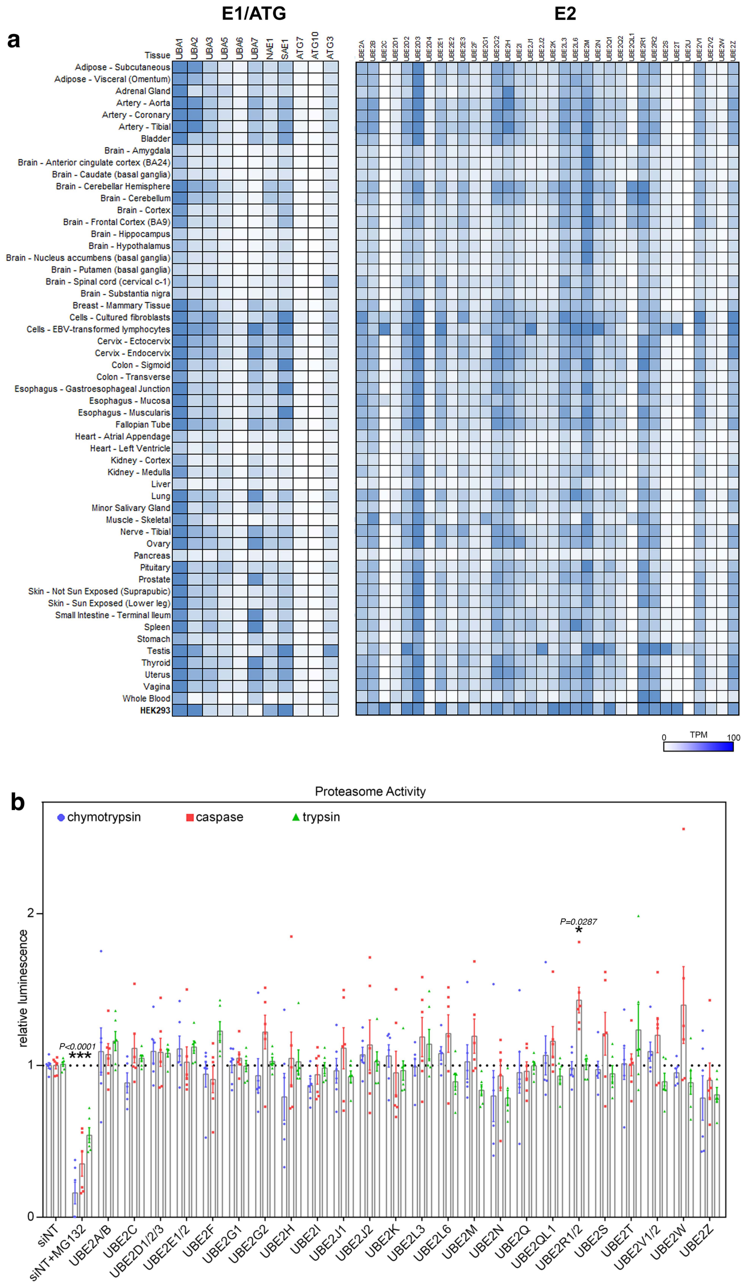

Supplementary Figure 1. E2s are widely expressed across human tissues and their knockdown does not impact proteasome activity. a Gene expression data retrieved from the GTEx database indicates that UBA1 and E2 ubiquitin-conjugating enzymes are widely expressed across human tissues, as well as in HEK293T cells. **b** No significant impact of E2 RNAi on proteasome activity in human HEK293 cells. Several E2s have been found to physically associate with the proteasome, and this could contribute to their capacity to modulate protein turnover. For example, UBE2D interacts with the proteasome in yeast, and this association further increases with heat stress, a condition where proteasome function is modulated by ubiquitination of proteasome components, such as Rpn13, via the proteasome-associated E3 UBE3C. To address whether E2s regulate proteasome activity, we have measured the chymotrypsin-like, caspase-like, and trypsin-like activities of the proteasome upon RNAi of individual or related E2s. As expected, proteasome inhibition by MG132 reduces the proteolytic activities of the proteasome. However, E2 knockdown does not significantly impact the activity of the proteasome, with the only exception of UBE2R1/2 knockdown, which leads to higher caspase-like activity, compared to control NT (non-targeting) siRNAs. The graphs report the mean ±SEM, with n=6 (biological replicates) and the *P*-values (two-way ANOVA, **P*<0.05 and ****P*<0.001). Source data are provided in the Source data file.

Proteomic changes induced by siRNAs for the indicated E2s. Proteins significantly modulated (P<0.05) with Log2FC>0.2 and -<0.2 independently from mRNA levels.

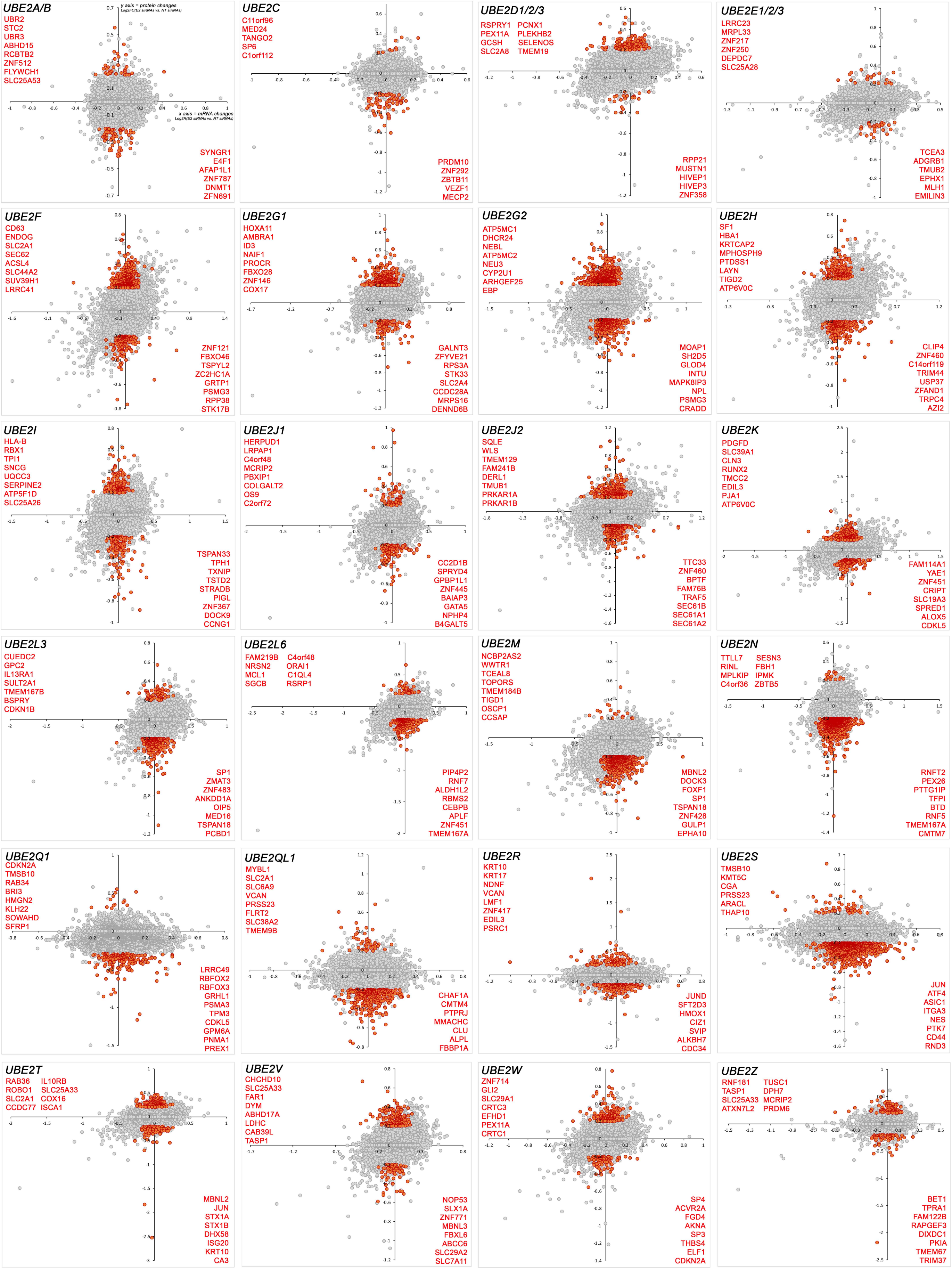

Supplementary Figure 2. Proteomic changes induced by siRNAs targeting individual and groups of related E2s. Deep-coverage TMT proteomics identifies proteins that are upregulated and downregulated by E2 RNAi in human HEK293T cells**.** Compared to control non-targeting (NT) siRNAs, knockdown of single or groups of related E2s leads to significant protein upregulation and downregulation (P<0.05; Log_2FC>0.2 and <-0.2; highlighted in red) and this does not result from corresponding changes in the mRNA levels. Some of the major upregulated and downregulated proteins are indicated for each E2 knockdown, compared to control non-targeting (NT) siRNAs. The *x*-axis displays the mRNA changes, log_2R(E2 vs. NT siRNAs), whereas the *y*-axis reports the protein changes, i.e. log_2FC(E2 vs. NT siRNAs). The proteomic data in this figure was obtained from 6 sets of 16-plex TMT analyses of E2 siRNAs (n=3/group), with each TMT set having its own set of control NT siRNAs (n=4). On average, each TMT set detected 10700 proteins: 5132 of these (mapping to 4676 DAVID IDs) were modulated by knockdown of one or more E2s in a mRNA-independent manner.
See also Supplementary Data 1.

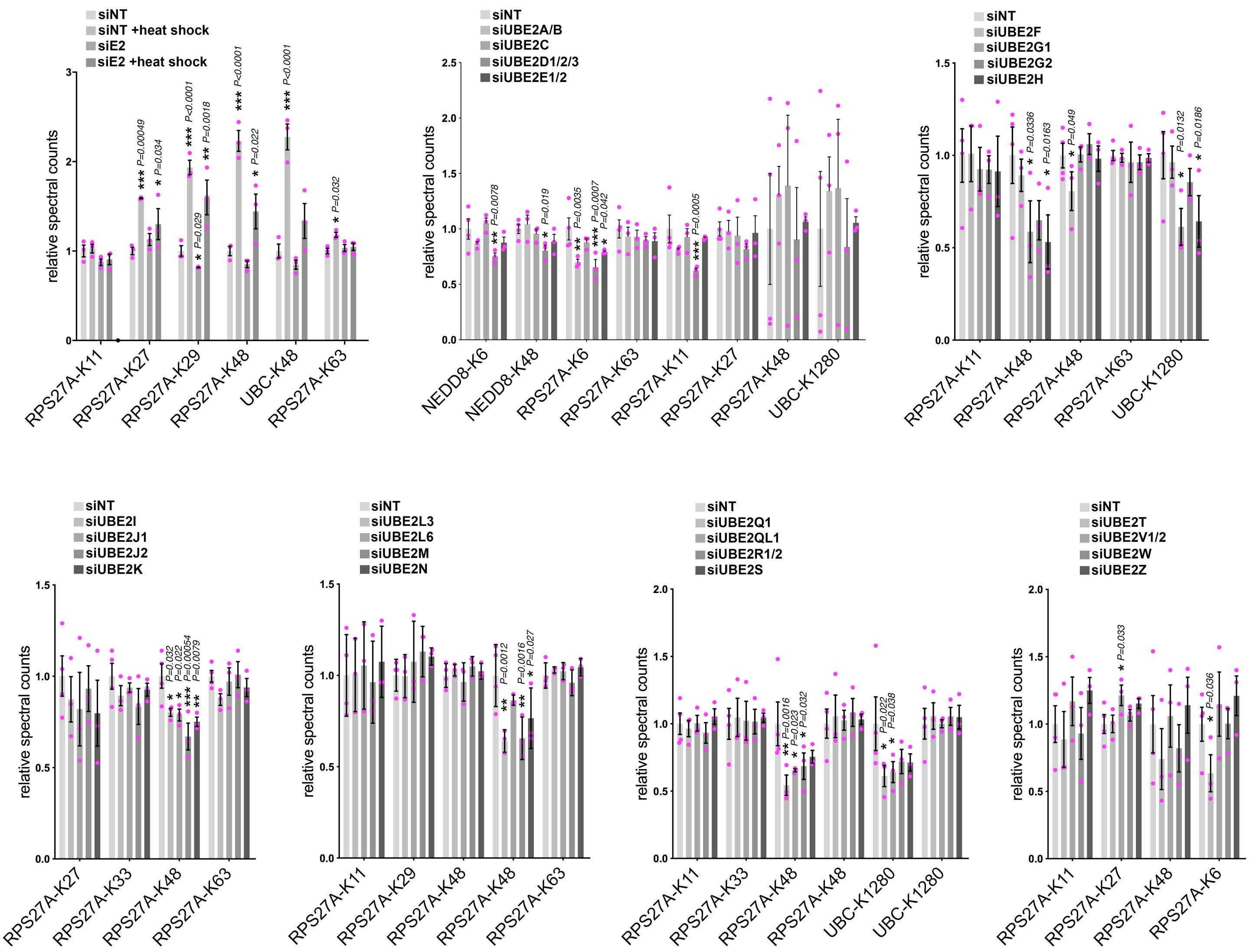

Supplementary Figure 3. JUMPptm identifies biases for linkage-specific ubiquitination by individual/related E2 ubiquitin-conjugating enzymes. Computational analysis of the TMT data obtained from HEK293T cells treated with siRNAs for individual and related E2 ubiquitin-conjugating enzymes, non-targeting (NT) control siRNAs, and E2combo siRNAs. The JUMPptm computational pipeline identifies changes in the linkage-specific ubiquitination of RPS27A, a fusion protein consisting of ubiquitin and the ribosomal protein S27A, which is cleaved to generate ubiquitin. This analysis is based on RPS27A (ubiquitin) because the specific linkage that JUMPptm identifies is on the ubiquitin protein: this represents the entirety of poly-ubiquitin (regardless of the substrate it is attached to) and indicates the linkage-specific bias of each E2 in building poly-ubiquitin chains. The graphs report the mean ±SEM, with n=3 (biological replicates) and the *P*-values (one-way ANOVA, **P*<0.05, ***P*<0.01, and ****P*<0.001). Source data are provided in the Source data file.
See also Supplementary Data 2.

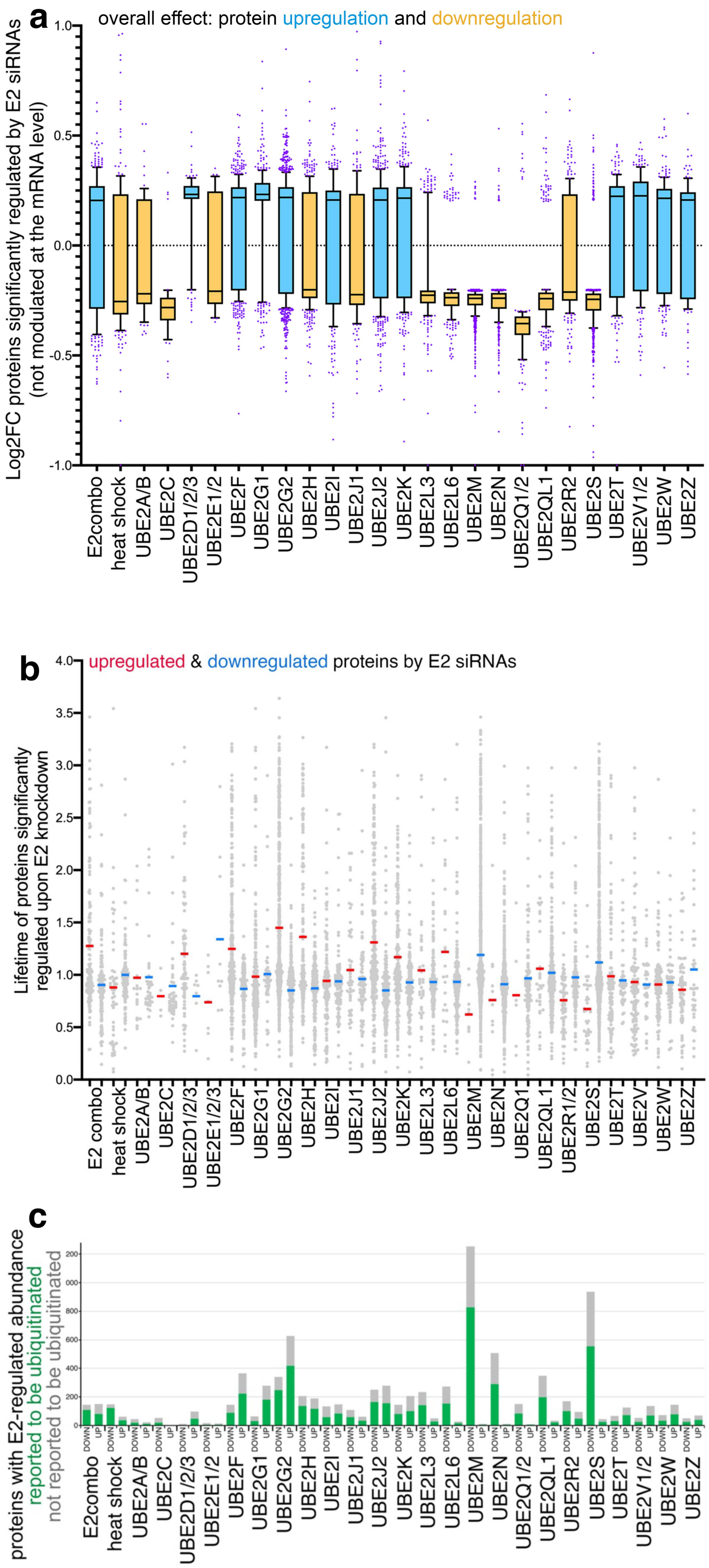
a
overall effect: protein upregulation and downregulation
Log2FC proteins significantly regulated by E2 siRNAs (not modulated at the mRNA level)
1.0
0.5
0.0
-0.5
-1.0
E2combo
heat shock
UBE2A/B
UBE2C
UBE2D1/2/3
UBE2E1/2
UBE2F
UBE2G1
UBE2G2
UBE2H
UBE2I
UBE2J1
UBE2J2
UBE2K
UBE2L3
UBE2L6
UBE2M
UBE2N
UBE2Q1/2
UBE2QL1
UBE2R2
UBE2S
UBE2T
UBE2V1/2
UBE2W
UBE2Z
b
upregulated & downregulated proteins by E2 siRNAs
Lifetime of proteins significantly regulated upon E2 knockdown
4.0
3.5
3.0
2.5
2.0
1.5
1.0
0.5
0.0
E2 combo
heat shock
UBE2A/B
UBE2C
UBE2D1/2/3
UBE2E1/2/3
UBE2F
UBE2G1
UBE2G2
UBE2H
UBE2I
UBE2J1
UBE2J2
UBE2K
UBE2L3
UBE2L6
UBE2M
UBE2N
UBE2Q1
UBE2QL1
UBE2R1/2
UBE2S
UBE2T
UBE2V
UBE2W
UBE2Z
c
proteins with E2-regulated abundance
reported to be ubiquitinated
not reported to be ubiquitinated
DOWN
UP
E2combo
heat shock
UBE2A/B
UBE2C
UBE2D1/2/3
UBE2E1/2
UBE2F
UBE2G1
UBE2G2
UBE2H
UBE2I
UBE2J1
UBE2J2
UBE2K
UBE2L3
UBE2L6
UBE2M
UBE2N
UBE2Q1/2
UBE2QL1
UBE2R2
UBE2S
UBE2T
UBE2V1/2
UBE2W
UBE2Z

Supplementary Figure 4. Proteins significantly regulated by E2 siRNAs are not enriched for short-lived proteins and include proteins previously reported to be ubiquitinated.

a Proteomic changes induced by siRNAs targeting individual and groups of related E2s. Protein changes (Log_2FC) significantly induced by E2 siRNAs ($P<0.05$; $Log_2FC>0.2$ and <-0.2) and not arising from corresponding mRNA changes. On average, the knockdown of some E2s leads primarily to protein downregulation (yellow) whereas other E2 siRNAs are biased towards protein upregulation (blue). Box plots and whiskers correspond to the 10-90 percentile.

b Proteins significantly regulated by E2 siRNAs are not enriched for short-lived proteins. A recent study (PMID:34626566) reports proteome-wide mapping of short-lived proteins in HEK293T cells. Short-lived proteins are characterized by a low ratio of protein abundance at 8h versus 0h from cycloheximide (CHX) treatment. Data from PMID:34626566 was extracted and compared to our proteomic surveys of E2-regulated proteins in HEK293T cells. Analysis of the lifetimes of proteins that are upregulated (red) and downregulated (blue) by E2 siRNAs. Protein changes significantly induced by E2 siRNAs ($P<0.05$; $Log_2FC>0.2$ and <-0.2) and not arising from corresponding mRNA changes are considered for this analysis. Red and blue lines represent the mean lifetimes of proteins that are upregulated and downregulated in response to the knockdown of the indicated E2; gray dots represent individual protein lifetime values. Overall, there is no enrichment for short-lived proteins among proteins regulated by E2 loss. However, albeit few, proteins that are upregulated upon RNAi for UBE2E1/2/3, UBE2M, UBE2N, and UBE2S have lifetimes (CHX 8h vs 0h) that trend towards lower values.

c Overlap between proteins with E2-regulated abundance and the ubiquitinome. A recent study (PMID:33431886) reports proteome-wide mapping of ubiquitinated proteins in HEK293 cells and identifies 5765 that are ubiquitinated. Proteins that we identify to have E2-regulated protein abundance and that are reported to be ubiquitinated in PMID:33431886 are shown in green whereas proteins for which ubiquitination has not been reported are shown in grey. Overall, ~64% of proteins with E2-regulated abundance were previously reported to be ubiquitinated in HEK293 cells.

Source data are provided in the Source data file.

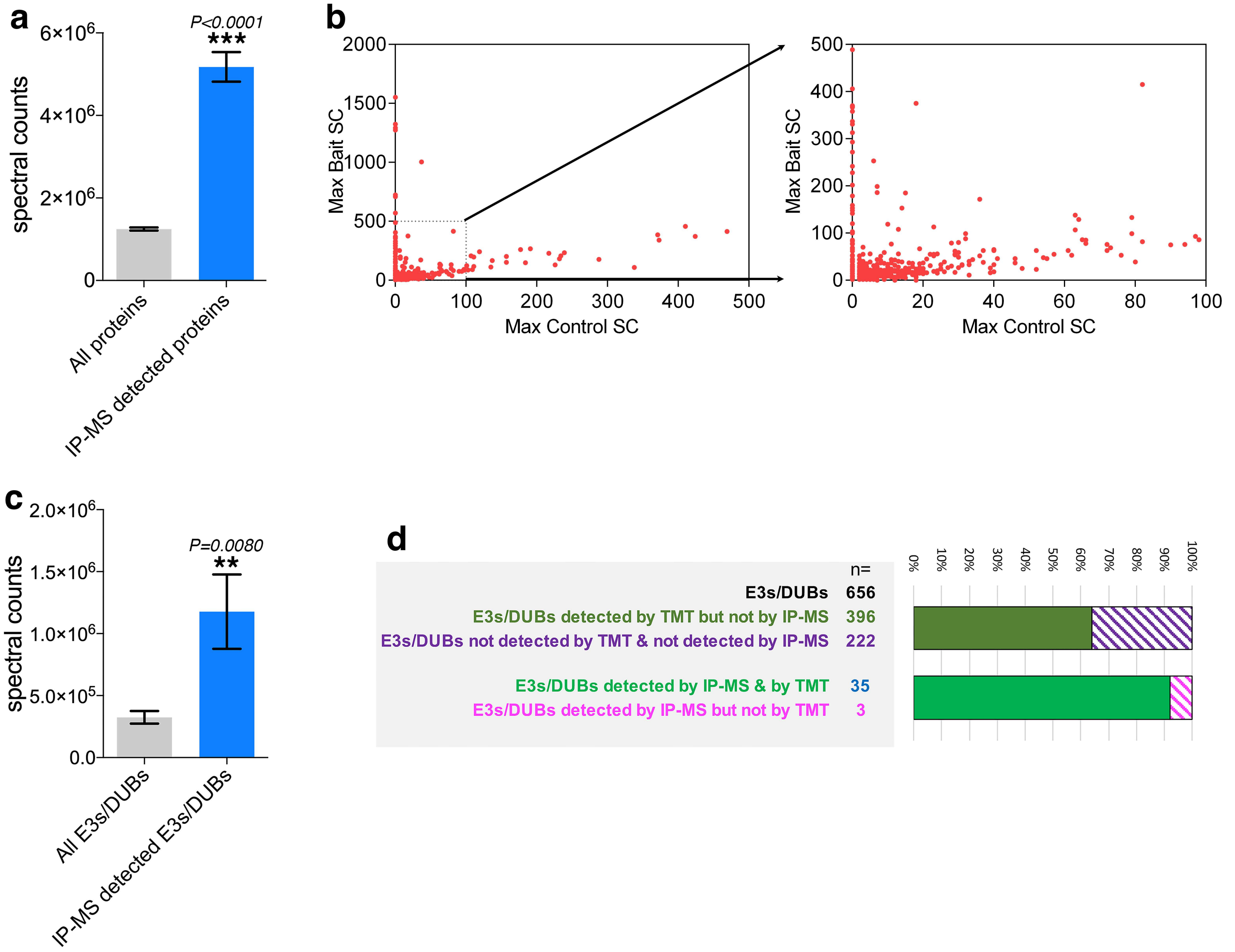

Supplementary Figure 5. Abundant E3s/DUBs are preferentially retrieved by IP-MS.
a The average spectral counts for proteins detected by affinity purification (IP-MS) is substantially higher than the average spectral counts for all TMT-detected proteins, indicating that poorly-expressed proteins might not be detected in these IP-MS experiments. The graph reports the mean ±SEM of the spectral counts for n=8851 (all proteins, TMT-detected) and n=434 (proteins retrieved by IP-MS); ****P*<0.0001 (two-tailed unpaired t-test with Welch's correction).
b The maximum spectral counts for proteins detected in association with E2 baits are consistently higher than those from control (no bait) purifications.
c The average spectral counts for E3 ubiquitin ligases (E3s) and deubiquitinating enzymes (DUBs) detected by IP-MS is substantially higher than the average spectral counts for all TMT-detected E3s and DUBs, indicating that poorly-expressed or transiently-interacting E3s/DUBs might not be detected in these IP-MS experiments. The graph reports the mean ±SEM of the spectral counts for n=396 (all E3s/DUBs, TMT-detected) and n=35 (E3s/DUBs retrieved by IP-MS); ***P*=0.0080 (two-tailed unpaired t-test with Welch's correction).
d E3s and DUBs that are not retrieved by IP-MS are enriched for poorly expressed E3s/DUBs that are not detected by TMT in HEK293T cells.
Source data are provided in the Source data file.

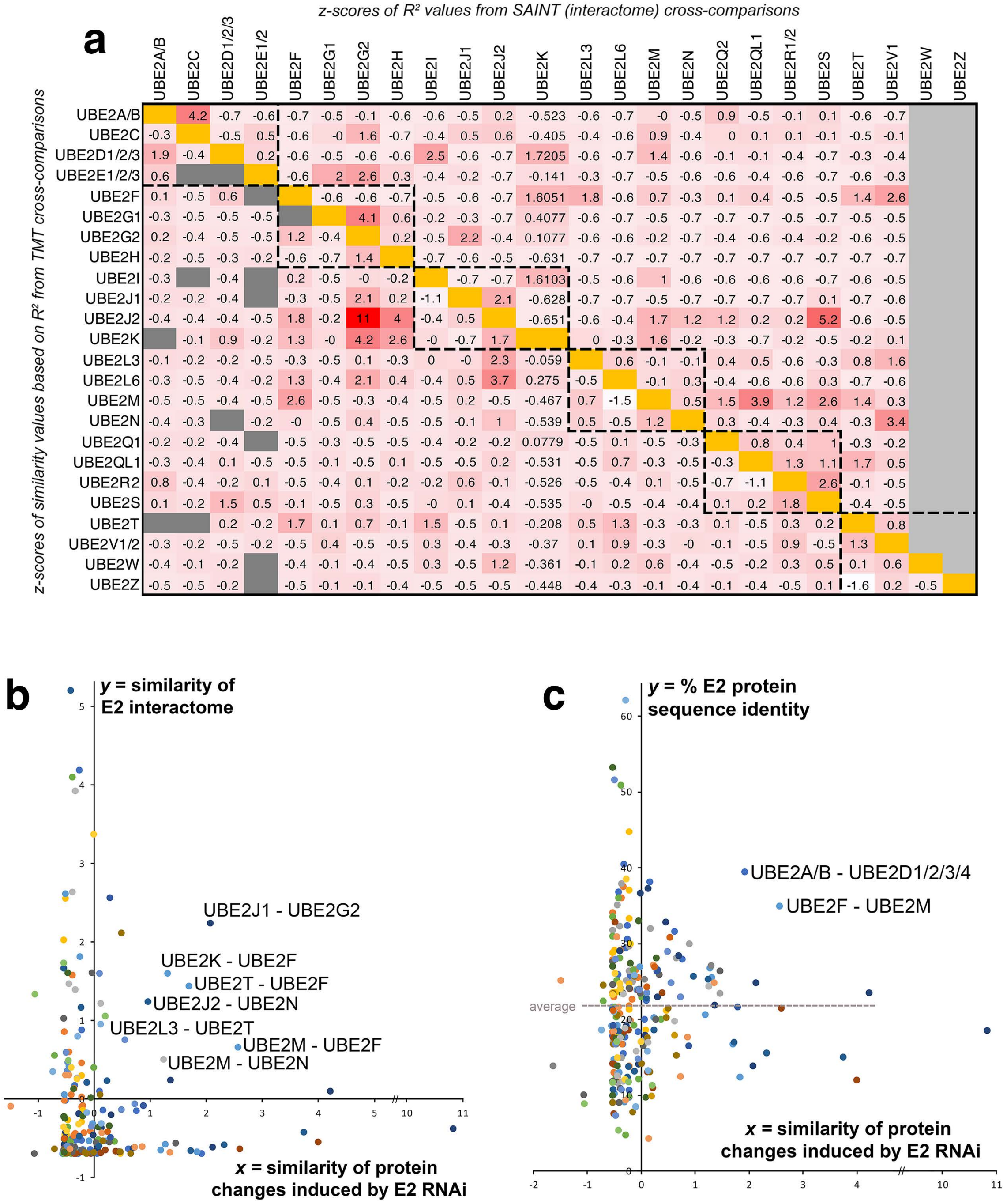

Supplementary Figure 6. Comparison of the similarity of the E2 physical interactome, E2 RNAi-induced protein changes, and % E2 protein identity.

a Comparison of the similarity of the physical interactome (z-score of the R^2 value that compares the SAINT score of E2x versus E2y) and RNAi-induced protein changes (z-score of the R^2 value from the cross-comparison of TMT datasets from E2x RNAi versus E2y RNAi, each normalized by its own NT RNAi control) for each E2 pair. Dashed boxes indicate E2 RNAi analyzed in the same TMT set.

b Similarity in the E2 interactome (*y*-axis) versus the protein changes induced by E2 RNAi (*x*-axis). There is overall little correlation for most E2 cross-comparisons, with some notable exceptions indicated.

c Cross-comparison of the % E2 protein identity (*y*-axis) with the similarity in the protein changes induced by E2 RNAi (*x*-axis) indicates that correlations found in (b) are not due to the sequence homology of the E2s in the pair, apart from UBE2M-UBE2F.

Source data are provided in the Source data file.

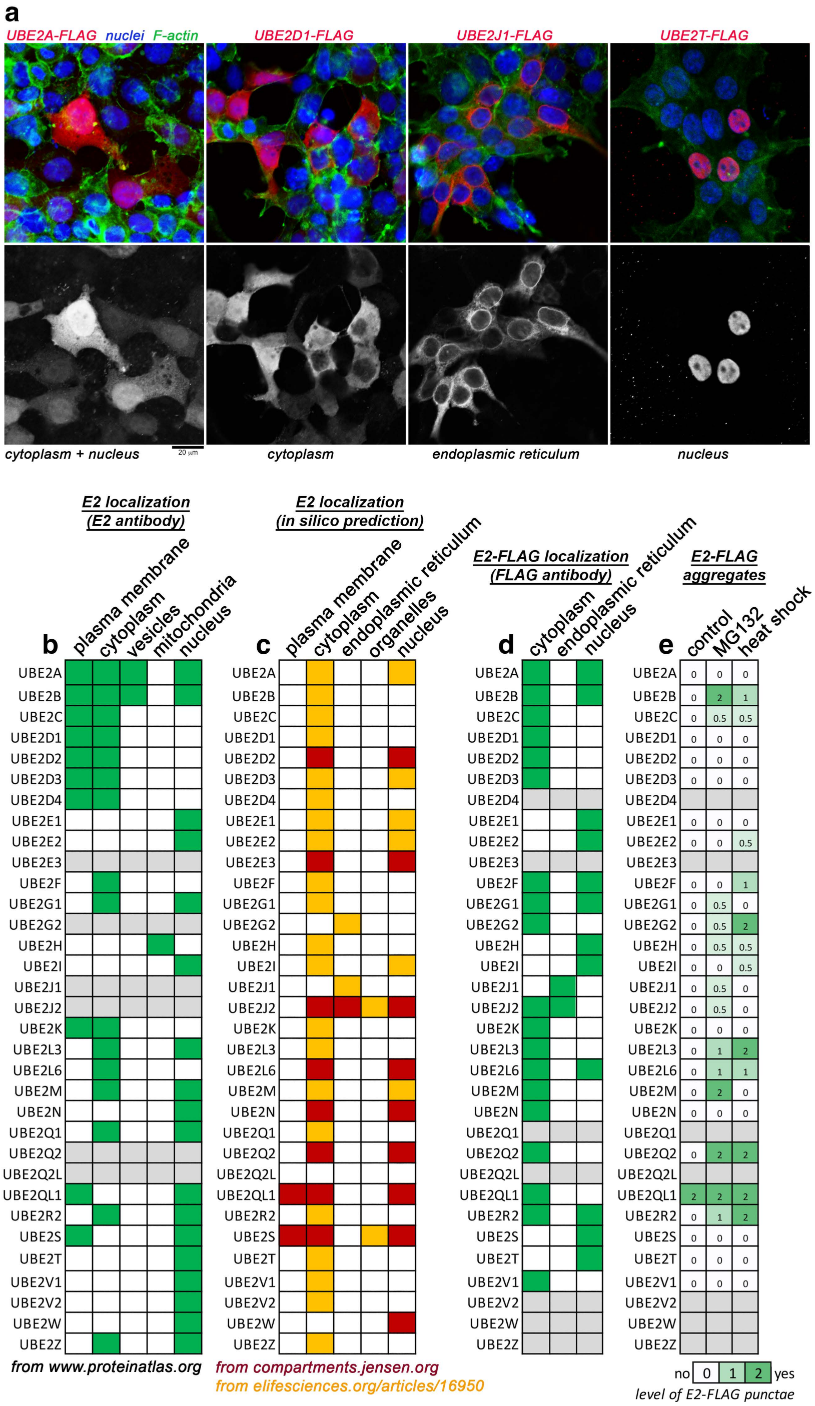

Supplementary Figure 7. Subcellular localization of endogenous and FLAG-tagged E2s.

a Representative images of HEK293T cells transfected with different FLAG-tagged E2s (red) and co-stained with DAPI and phalloidin to identify nuclei (blue) and F-actin (green); scale bar, 20 μm. UBE2A-FLAG is detected in the cytoplasm and nucleus, UBE2D1-FLAG in the cytoplasm, UBE2J1-FLAG in the endoplasmic reticulum, and UBE2T-FLAG in the nucleus. Similar analyses have been done for all FLAG-tagged E2s (d) used to build the E2 interactome and are reported in (d).

b-d The subcellular localization of FLAG-tagged E2s is overall similar to the localization of endogenous E2s as detected with antibody immunostaining (b; data retrieved from the Protein Atlas) and also based on in silico predictions (c; from compartments.jensen.org) and proteomic mapping (c; from PMID:27278775).

e Some FLAG-tagged E2s localize to protein aggregates upon proteasome inhibition (MG132) and heat shock (green) compared to controls (0=no aggregates; 1=some aggregates; 2= aggregates).

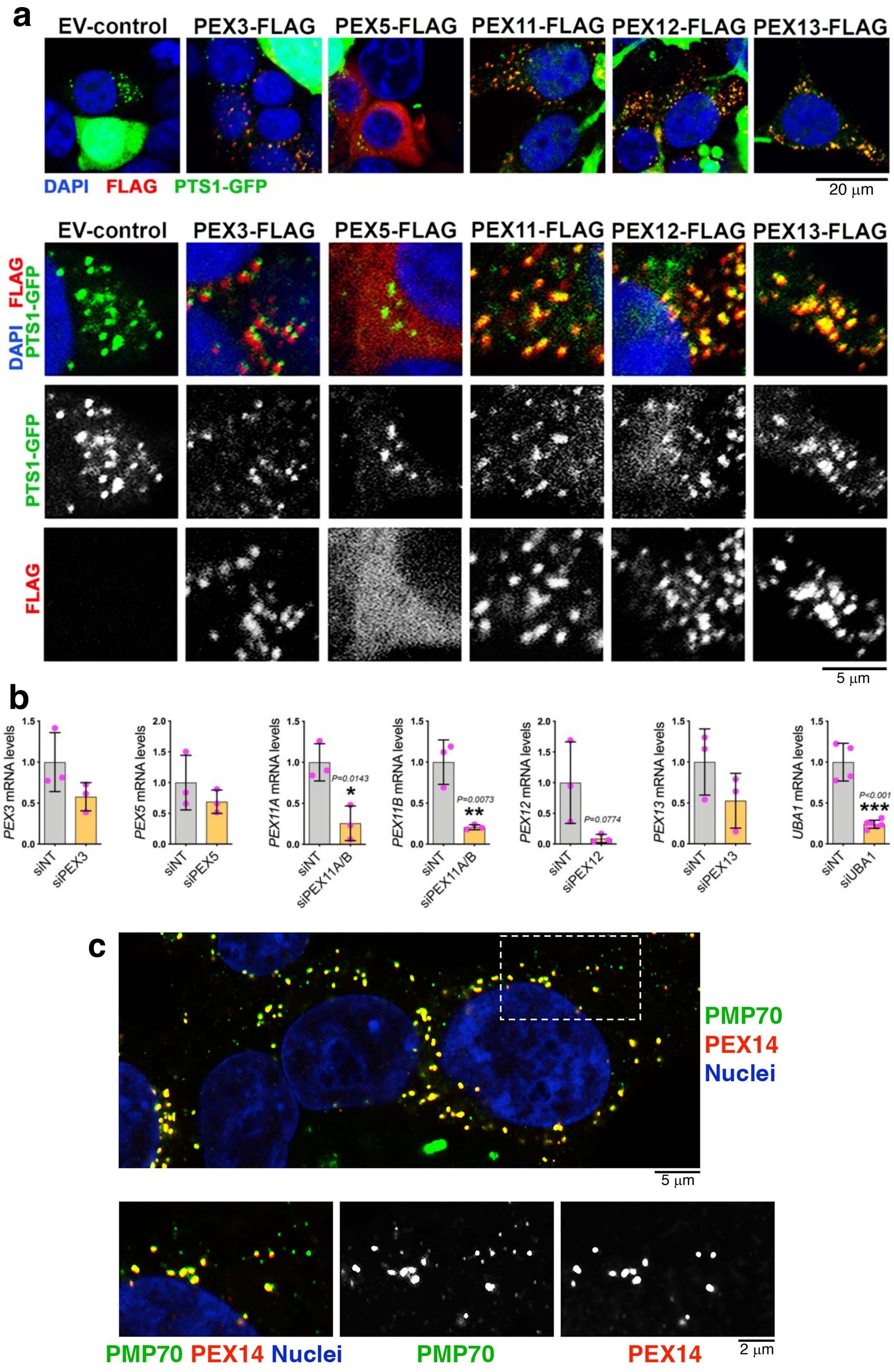

Supplementary Figure 8. Validation of FLAG-tagged PEX proteins and PEX-targeting siRNAs.
a FLAG-tagged PEX proteins localize to functional peroxisomes (identified by GFP-PTS1) in HEK293T cells, as indicated by the co-localization (yellow) of PEX-FLAG (red) and GFP-PTS1 (green). In addition to peroxisomes, PEX5-FLAG also localizes to the cytoplasm, consistent with its known role in importing peroxisomal proteins into the peroxisomal matrix, which requires PEX5 shuttling between the peroxisomal membrane and the cytoplasm. DAPI staining is also shown in blue; EV=empty vector control.
b Validation of siRNAs for PEX proteins. Knockdown of PEX proteins via siRNAs reduces PEX mRNA levels compared to control NT siRNAs; n=3 (biological replicates) with the mean ±SD and *P*-values (unpaired two-tailed t-test) indicated (**P*<0.05, ***P*<0.01, and ****P*<0.001).
c Immunostaining for PMP70 and PEX14 largely overlap in HEK293T cells.
Source data are provided in the Source data file.

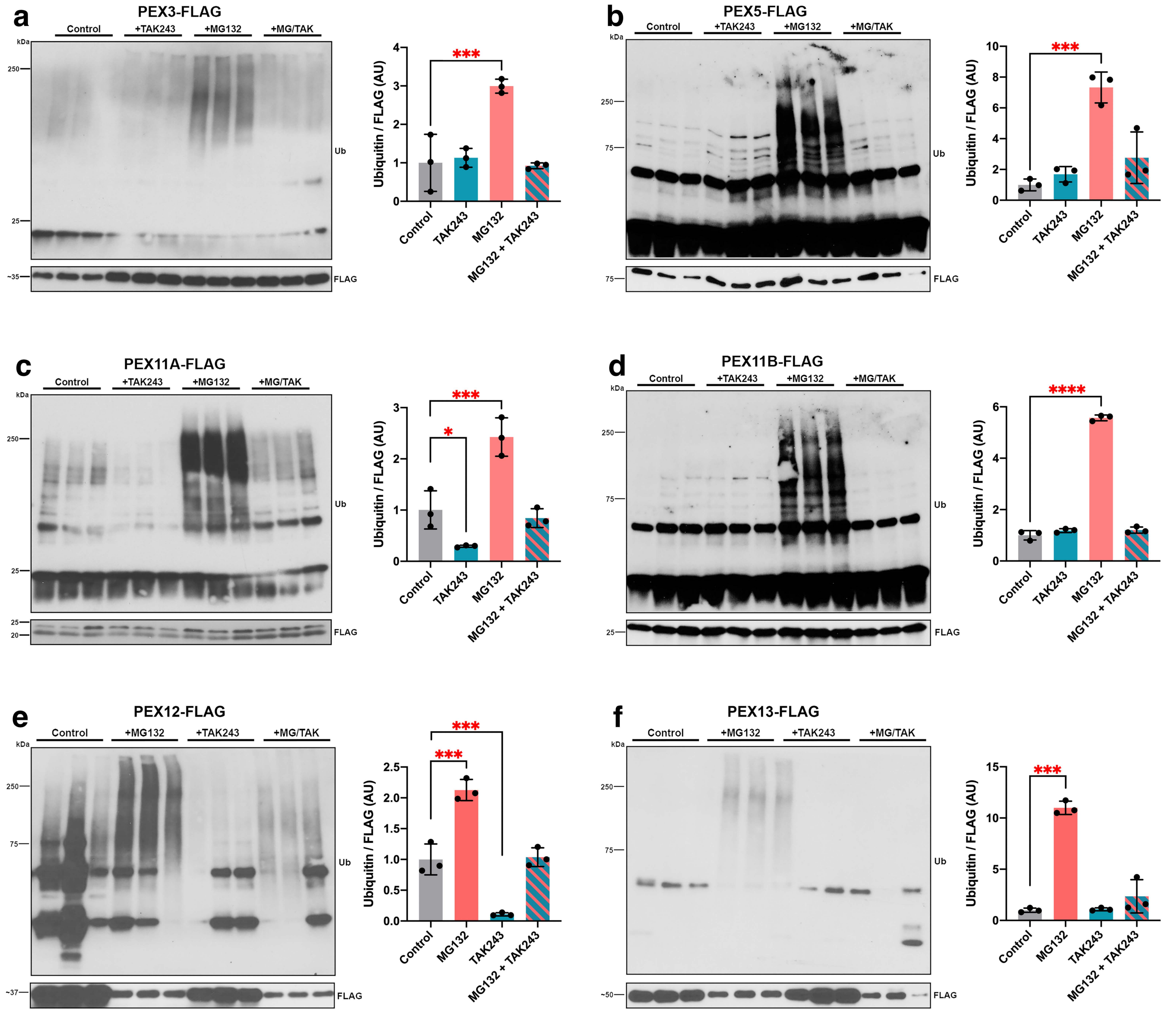

Supplementary Figure 9. PEX turnover occurs at least in part via the proteasome.
a-f Western blots related to Figure 4f. Immunoprecipitation of FLAG-tagged versions of some of the PEX proteins that are upregulated by UBA1/E2combo RNAi. Anti-ubiquitin (Ub) immunoblotting is used to determine the ubiquitination status of FLAG-tagged PEX proteins. Compared to controls (grey), treatment with the proteasome inhibitor MG132 (orange) increases PEX ubiquitination and this is largely prevented by concomitant treatment with the UBA1 inhibitor TAK243 (orange-green stripes). Similar results are obtained for PEX3-FLAG (a), PEX5-FLAG (b), PEX11A-FLAG (c), PE11B-FLAG (d), PEX12-FLAG (e), and PEX13-FLAG (f); mean ±SD with n=3 and *P* values (one-way ANOVA) indicated (**P*<0.05, ****P*<0.001). Figure 4f reports the precise *P*-values. These findings indicate that peroxin turnover occurs at least in part via the proteasome. The lower bands (~25 and ~50 kDa) present in some of the lanes may derive from small amounts of resin with anti-Flag IgG that gets loaded on the gel (this does not interfere with the measurement of poly-ubiquitin and FLAG-tagged proteins). Source data are provided in the Source data file.

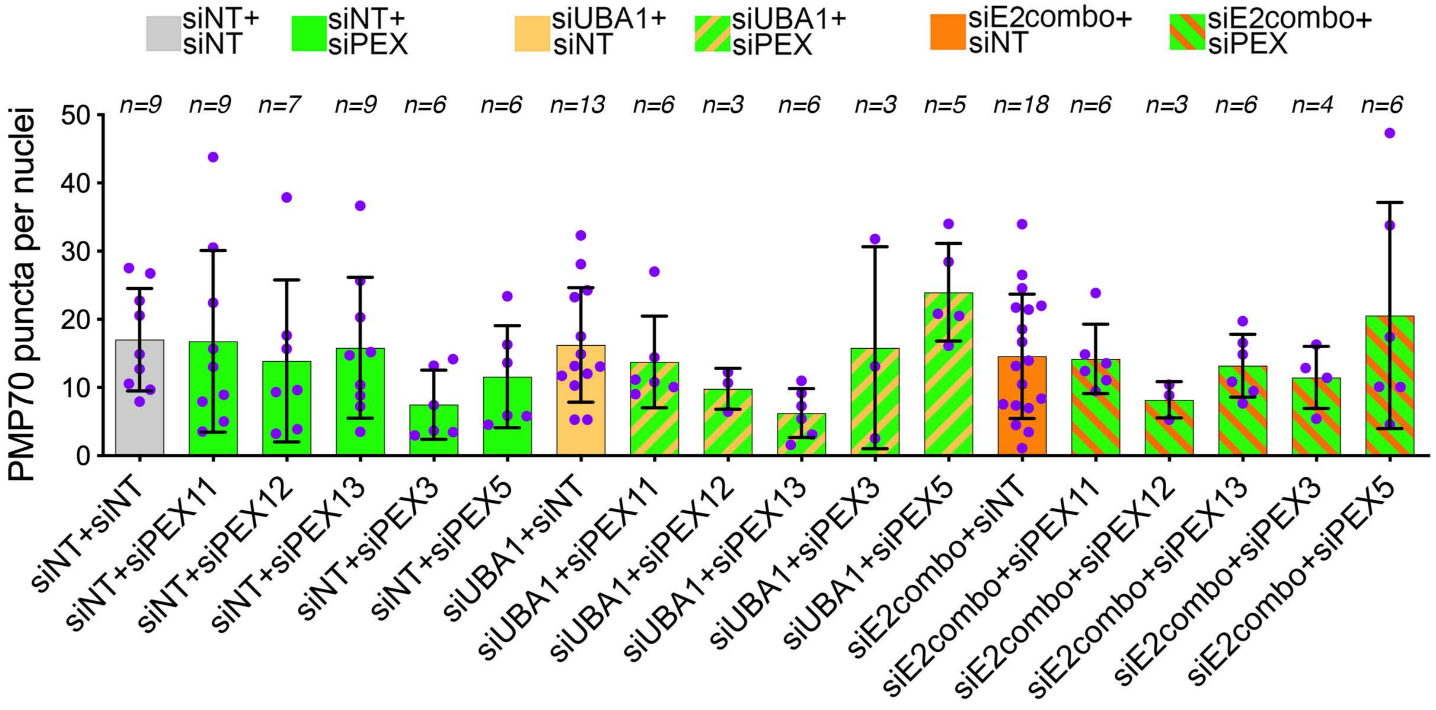

Supplementary Figure 10. Knockdown of UBA1 and E2combo does not substantially impact the number of peroxisomes compared to NT siRNA controls.

The average number of peroxisomes was defined based on the number of PMP70-positive puncta normalized by the number of nuclei in a cell population. Each replicate corresponds to a biologically independent cell culture well. All comparisons are not significant (*P*>0.05, one-way ANOVA), with the mean ±SD and n indicated in the figure. Altogether, these analyses indicate that the knockdown of UBA1 and E2combo does not significantly impact the number of peroxisomes compared to NT controls. Source data are provided in the Source data file.

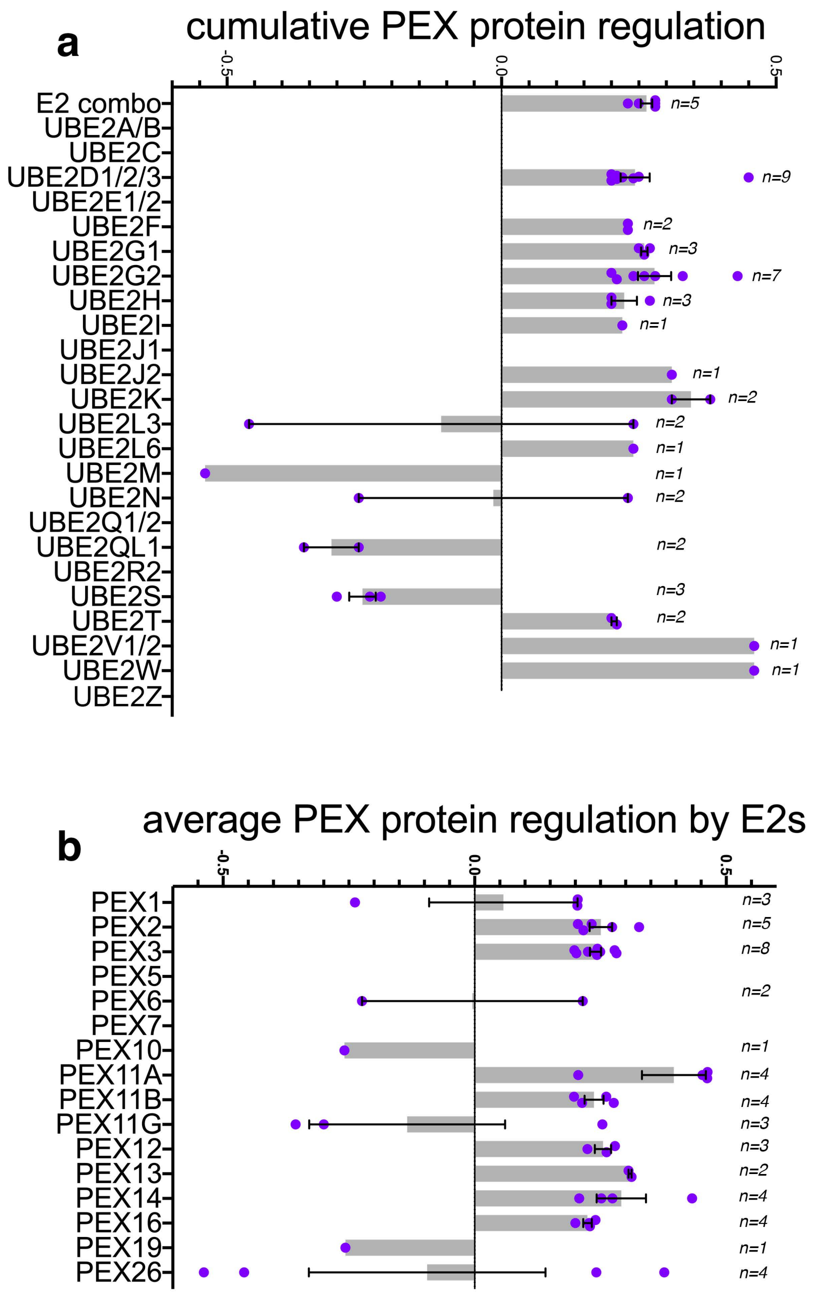

Supplementary Figure 11. Cumulative regulation of PEX proteins by multiple E2 ubiquitin-conjugating enzymes. a Modulation of the levels of multiple PEX proteins by siRNAs targeting individual and related E2s. Each dot corresponds to a different PEX protein modulated by the specific E2 siRNA intervention indicated on the left. The mean ±SEM and n are indicated in the figure. **b** Modulation of each PEX protein (indicated on the left) by siRNAs targeting individual and related E2s. Each dot corresponds to a different E2 siRNA intervention that significantly modulates PEX protein levels. The mean ±SEM and n are indicated in the figure.
Related to Fig. 6a-b. Source data are provided in the Source data file.

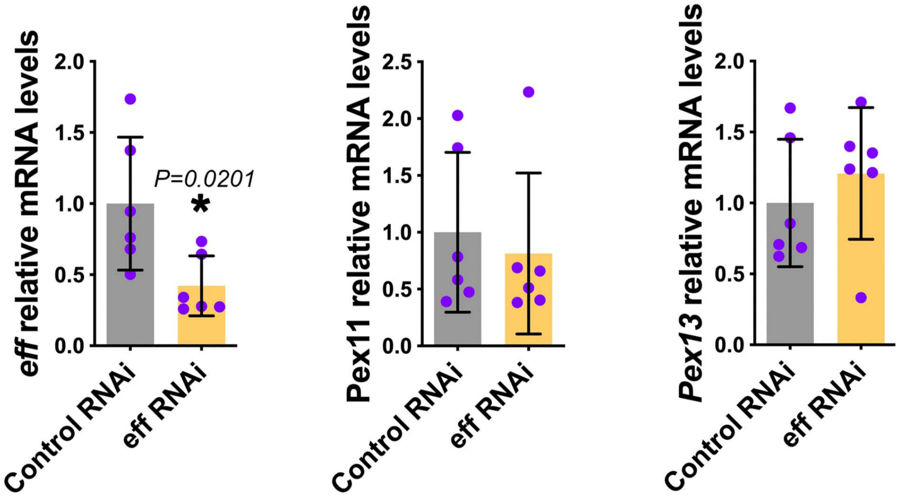

Supplementary Figure 12. UBE2D/eff knockdown does not regulate Pex11 and Pex13 mRNA levels in *Drosophila* skeletal muscles. Knockdown of eff/UBE2D significantly reduces eff mRNA levels but does not impact Pex11 and Pex13 mRNA levels; n=6 (biological replicates) with the mean ±SD and *P*-values (unpaired two-tailed t-test) indicated (**P*<0.05).
Source data are provided in the Source data file.
