# Supplementary material for: An adaptive stress response that confers cellular resilience to decreased ubiquitination
Source: Nat Commun. 2023 Nov 14;14:7348. doi: 10.1038/s41467-023-43262-7 (PMC10646096; doi:10.1038/s41467-023-43262-7)
Supplement: Supplementary file 3 — Description of Additional Supplementary Files [file 41467_2023_43262_MOESM3_ESM.pdf]

Description of Additional Supplementary Files

Supplementary Data 1. TMT proteomics data of HEK293T cells with knockdown of individual or related E2s, and of *Drosophila* skeletal muscle with knockdown of eff/UBE2D, rescue with human UBE2D2, and controls.

Supplementary Data 2. Analysis of TMT proteomics datasets with JUMPptm to identify the linkage-specific bias of E2s in building poly-ubiquitin chains.

Supplementary Data 3. E1 spectral counts and SAINT analysis of IP-MS experiments.

Supplementary Data 4. E2 spectral counts and SAINT analysis of IP-MS experiments.

Supplementary Data 5. Sequences of the oligos utilized for qRT-PCR in human cells and in *Drosophila*.

Supplementary Data 6. Sequences of the oligos utilized for cloning E1s and E2s to generate pCDH-EF1-E1/E2-FLAG-STOP (T2A-GFP) plasmids used as baits in IP-MS experiments.
